# Supplementary material for: Frustrated endocytosis controls contractility-independent mechanotransduction at clathrin-coated structures
Source: Nat Commun. 2018 Sep 20;9:3825. doi: 10.1038/s41467-018-06367-y (PMC6148028; doi:10.1038/s41467-018-06367-y)
Supplement: Supplementary file 1 — Supplementary Information [file 41467_2018_6367_MOESM1_ESM.pdf]

Supplementary-information

Baschieri *et al.*

Title: Frustrated endocytosis controls contractility-independent mechanotransduction at clathrin-coated structures

a

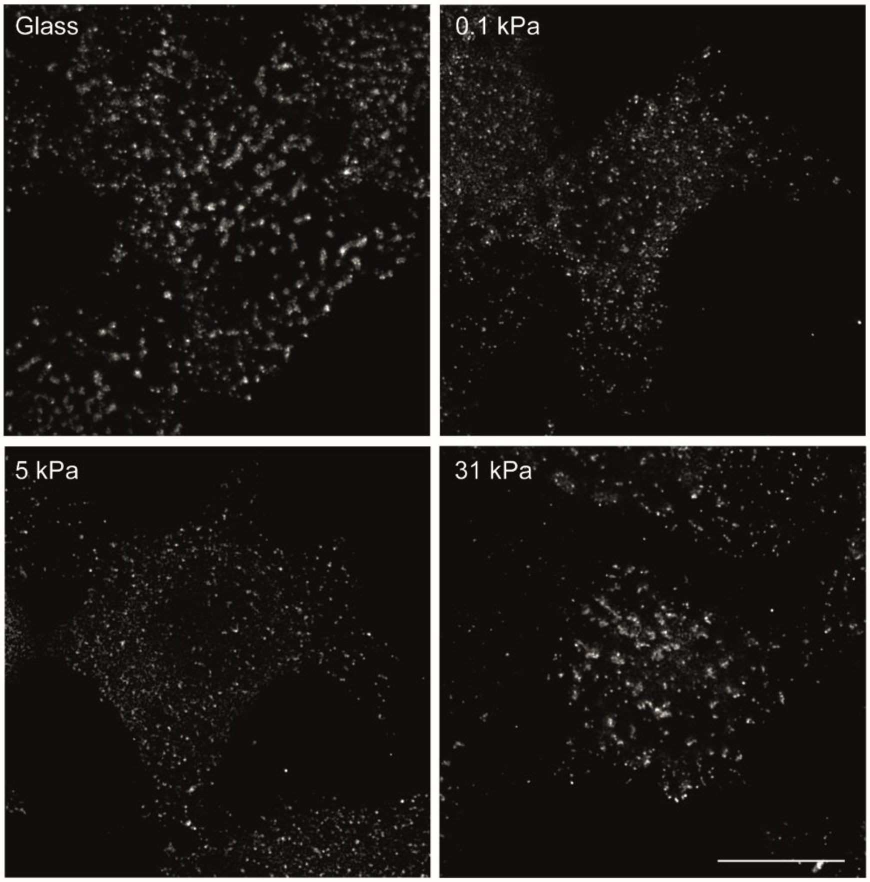

b

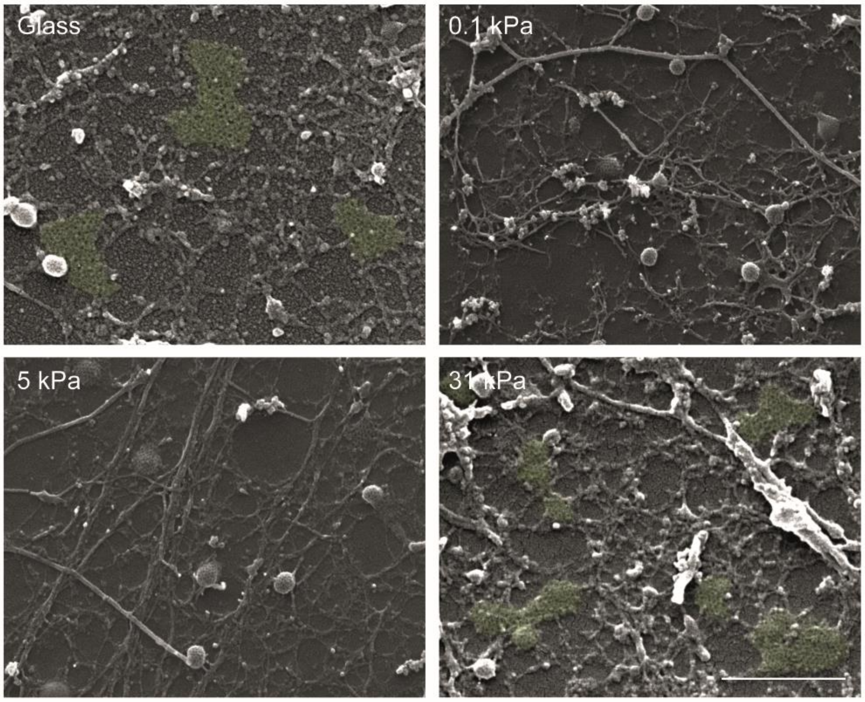

SUPPLEMENTARY FIGURE 1. **High resolution analysis of elasticity-dependent plaque formation. a**, HeLa cells were seeded on collagen-coated glass or collagen-coated polyacrylamide gels of indicated stiffness and fixed 24h later before being stained for α-adaptin and imaged by super resolution, stimulated-emission-depletion (STED) microscopy. Scale bar: 5 µm. **b**, EM micrographs of unroofed HeLa cells that were cultured on glass or polyacrylamide gels of indicated stiffness before being fixed and processed. Clathrin-coated plaques are highlighted in green. Scale bar: 300 nm.

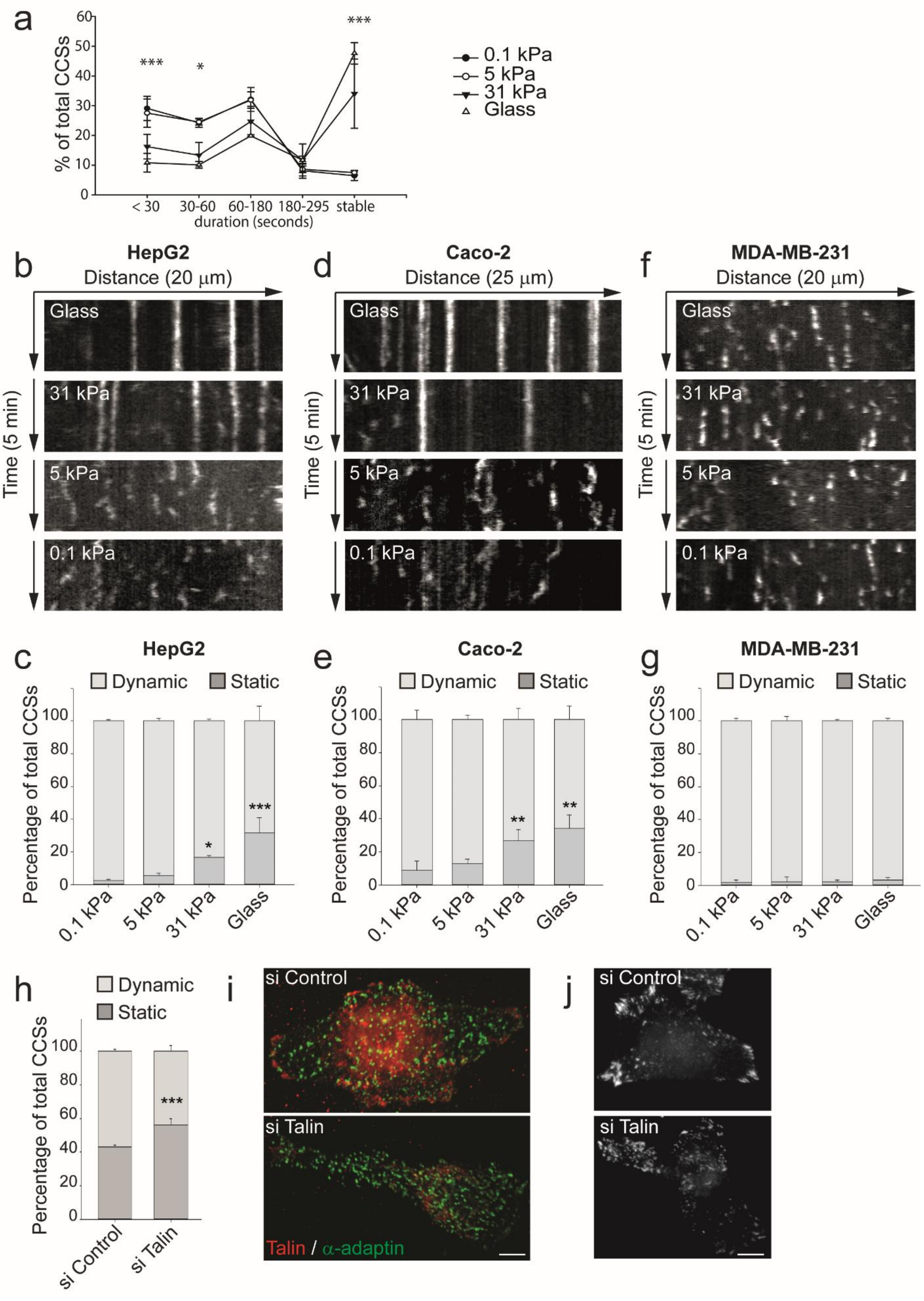

a
% of total CCSs
0.1 kPa
5 kPa
31 kPa
Glass
***
*
***
< 30
30-60
60-180
180-295
stable
duration (seconds)
b
HepG2
Distance (20 μm)
Time (5 min)
Glass
31 kPa
5 kPa
0.1 kPa
d
Caco-2
Distance (25 μm)
f
MDA-MB-231
Distance (20 μm)
c
HepG2
e
Caco-2
g
MDA-MB-231
Dynamic
Static
Percentage of total CCSs
h
si Control
si Talin
i
Talin / α-adaptin
j

SUPPLEMENTARY FIGURE 2. **Analysis of stiffness-dependent CCS dynamics regulation in different cell types.** **a**, Distribution of CCS lifetimes on substrates of different stiffness coated with collagen (*** $P<0.001$, * $P<0.05$ comparing glass to 0.1 kPa, as assessed by One Way Analysis of Variance – ANOVA. N=3). **b**, Kymographs showing CCS dynamics in HepG2 cells transfected with µ2-adaptin-GFP, seeded on collagen-coated glass or on collagen-coated polyacrylamide gels of the indicated stiffness, and imaged by spinning disk microscopy every 5s for 5 min. **c**, Quantification of the dynamics of CCSs observed as in b (*** $P<0.001$, * $P<0.05$, as compared to the 0.1 kPa condition, as assessed by One Way Analysis of Variance – ANOVA. N=3). **d**, Kymographs showing CCS dynamics in Caco-2 cells transfected with µ2-adaptin-GFP, seeded on collagen-coated glass or on collagen-coated polyacrylamide gels of the indicated stiffness, and imaged by spinning disk microscopy every 5s for 5 min. **e**, Quantification of the dynamics of CCSs observed as in d (** $P<0.005$, * $P<0.05$, as compared to the 0.1 kPa condition, as assessed by One Way Analysis of Variance – ANOVA. N=3). **f**, Kymographs showing CCS dynamics in genome-edited MDA-MB-231 cells expressing µ2-adaptin-GFP, seeded on collagen-coated glass or on collagen-coated polyacrylamide gels of the indicated stiffness, and imaged by spinning disk microscopy every 5s for 5 min. **g**, Quantification of the dynamics of CCSs observed as in f (One Way Analysis of Variance – ANOVA. N=3) **h**, Quantification of CCS dynamics in genome-edited HeLa cells expressing µ2-adaptin-GFP, treated with a Talin1-specific siRNA, seeded on collagen-coated glass, and imaged by spinning disk microscopy every 5s for 5 min. (*** $P<0.001$, as assessed by One Way Analysis of Variance – ANOVA. N=3). **i**, HeLa cells treated with control or Talin1-specific siRNA, fixed and immunostained for α-adaptin and Talin1. Scale bar 5 µm. **j**, HeLa cells treated with control or Talin1-specific siRNA were fixed and immunostained for Vinculin. Scale bar 5 µm. All data are expressed as mean ± SD.

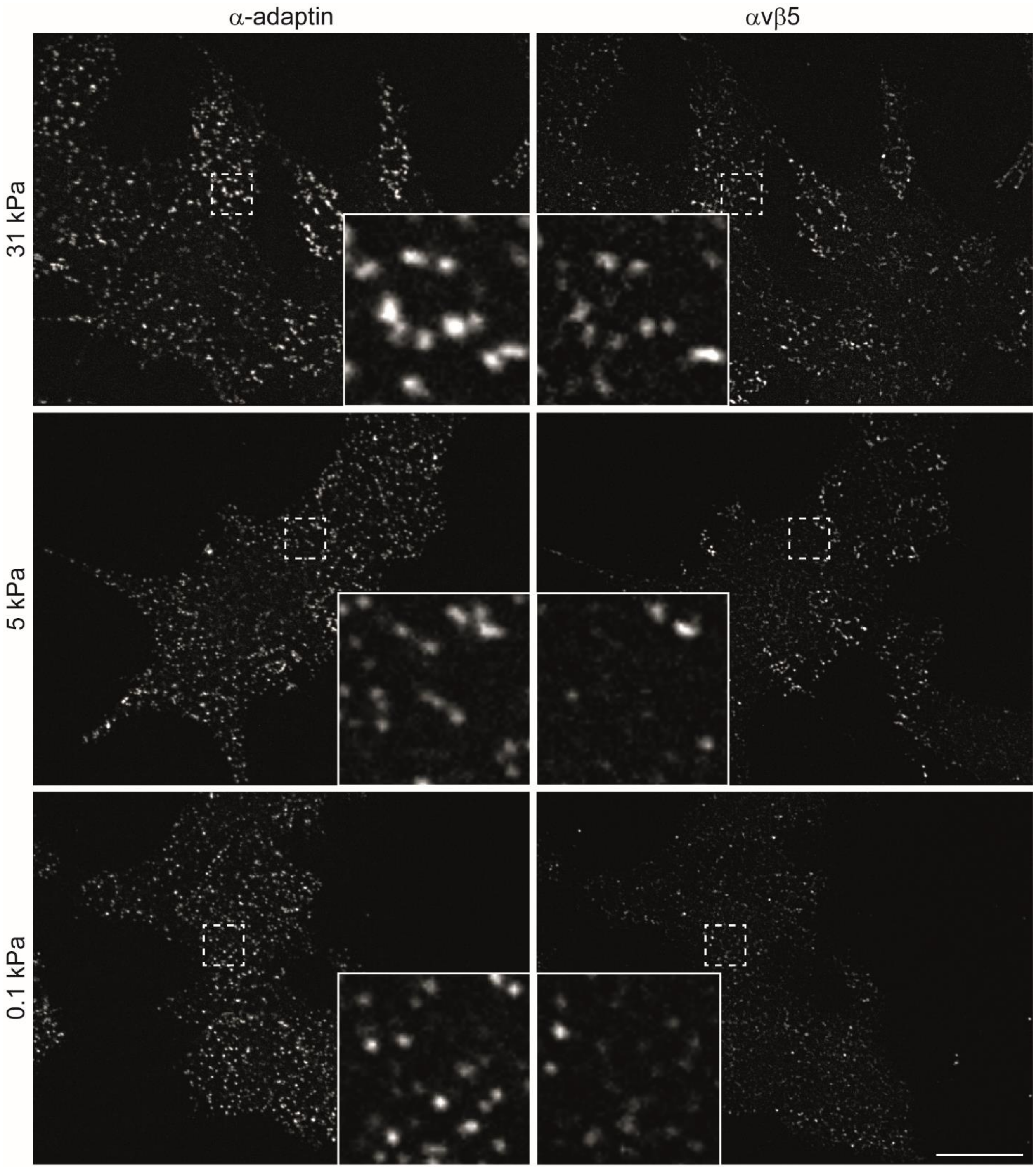

SUPPLEMENTARY FIGURE 3. **Integrin αvβ5 localizes to clathrin-coated plaques on stiff substrates**. HeLa cells were seeded on collagen-coated polyacrylamide gels of indicated stiffness and fixed 24h later before being stained for α-adaptin and αvβ5 and imaged by spinning disk microscopy. Scale bar: 15 µm. Magnifications of boxed regions are shown.

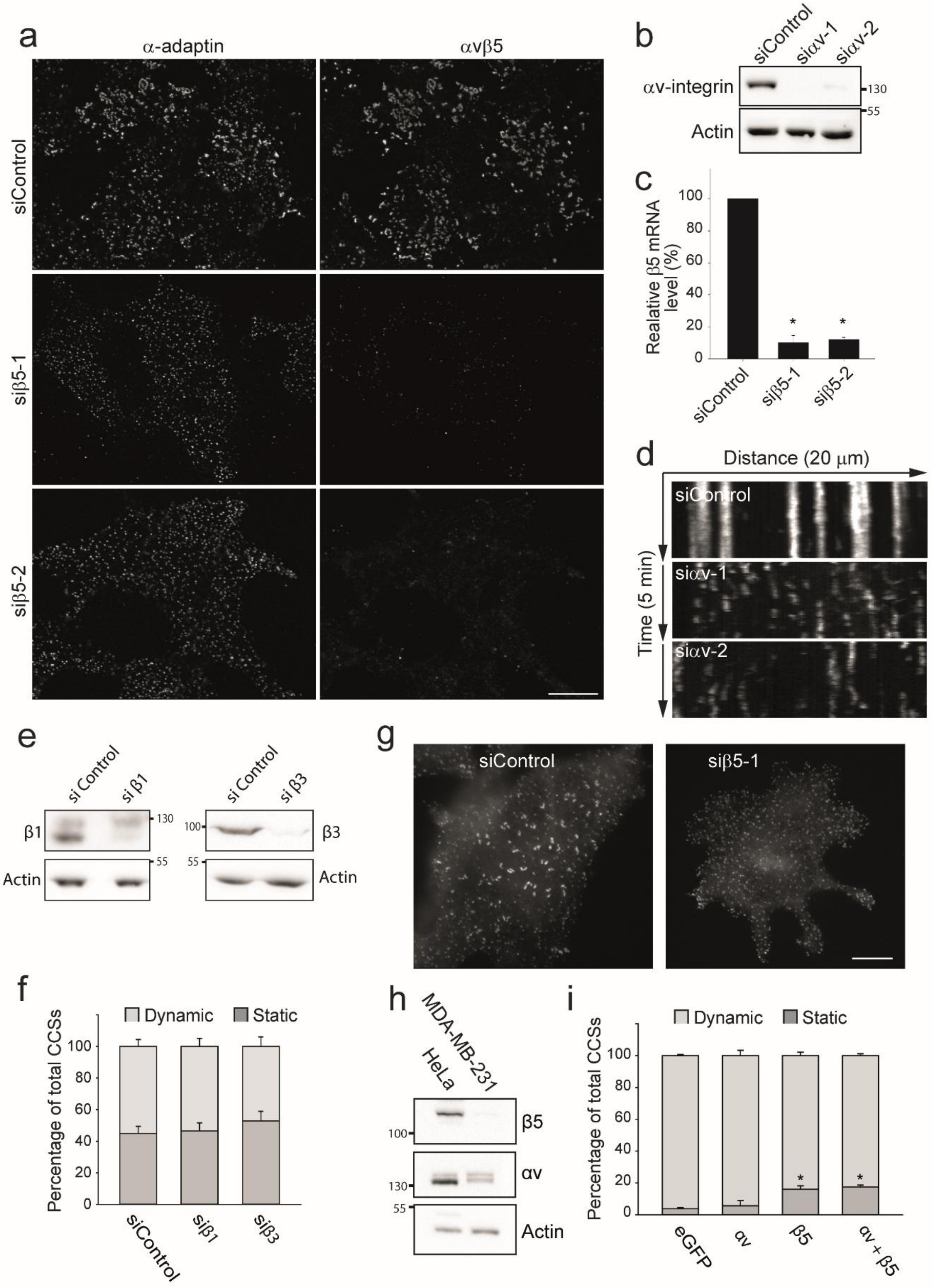

a
α-adaptin
αvβ5
siControl
siβ5-1
siβ5-2
b
siControl
siαv-1
siαv-2
αv-integrin
130
55
Actin
c
Realative β5 mRNA level (%)
100
80
60
40
20
0
siControl
siβ5-1
siβ5-2
d
Distance (20 μm)
Time (5 min)
siControl
siαv-1
siαv-2
e
si Control
si β1
β1
130
55
Actin
si Control
si β3
100
55
β3
Actin
g
siControl
siβ5-1
f
Dynamic
Static
Percentage of total CCSs
100
80
60
40
20
0
siControl
siβ1
siβ3
h
MDA-MB-231
HeLa
β5
100
αv
130
55
Actin
i
Dynamic
Static
Percentage of total CCSs
100
80
60
40
20
0
eGFP
αv
β5
αv + β5

SUPPLEMENTARY FIGURE 4. **Clathrin-coated plaque assembly depends on integrin αvβ5**. **a**, Genome-edited HeLa cells treated with control (upper panel) or β5-specific (lower panels) siRNAs for 48h were seeded on collagen-coated glass and fixed 24h later before being immunostained for α-adaptin and αvβ5 and imaged by spinning disk microscopy. Scale bar: 15 µm. **b**, Western-blot analysis of integrin αv expression in HeLa cells treated with the indicated siRNAs for 72h. Actin was used as a loading control (representative image of three independent experiments) **c**, Relative Integrin β5 mRNA levels in HeLa cells treated with the indicated siRNAs for 72h. (* $P<0.001$, as assessed by One Way Analysis of Variance – ANOVA. N=3). **d**, Kymographs showing CCS dynamics in genome-edited HeLa cells that were treated with control or αv-specific siRNAs for 48h, seeded on collagen-coated glass and imaged 24h later by spinning disk microscopy every 5s for 5 min. **e**, Western-blot analysis of integrin β1 (left panel) and integrin β3 (right apnel) expression in HeLa cells treated with the indicated siRNAs for 72h. Actin was used as a loading control. **f**, Quantification of CCS dynamics in genome-edited HeLa cells expressing µ2-adaptin-GFP, treated with the indicated siRNAs, seeded on collagen-coated glass, and imaged by spinning disk microscopy every 5s for 5 min (One Way Analysis of Variance – ANOVA. N=3) **g**, HepG2 cells treated with control (left panel) or β5 specific (right panel) siRNA for 48h were plated on collagen-coated glass and fixed 24h later before being stained for α-adaptin and imaged by epifluorescence microscopy. Scale bar: 5 µm. **h**, HeLa and MDA-MB-231 cells were lysed and analysed by western blot for integrin-β5 and integrin-αv. Actin was used as loading control (representative image of three independent experiments) **i**, MDA-MB-231 cells genome-edited to express RFP-tagged µ2-adaptin were transfected with plasmids encoding the indicated proteins, then seeded on vitronectin-coated glass and imaged by spinning disk microscopy every 5s for 5 min in order to quantify CCSs dynamics. (* $P<0.001$ as compared to eGFP, as assessed by One Way Analysis of Variance – ANOVA. N=3). All data are expressed as mean ± SD.

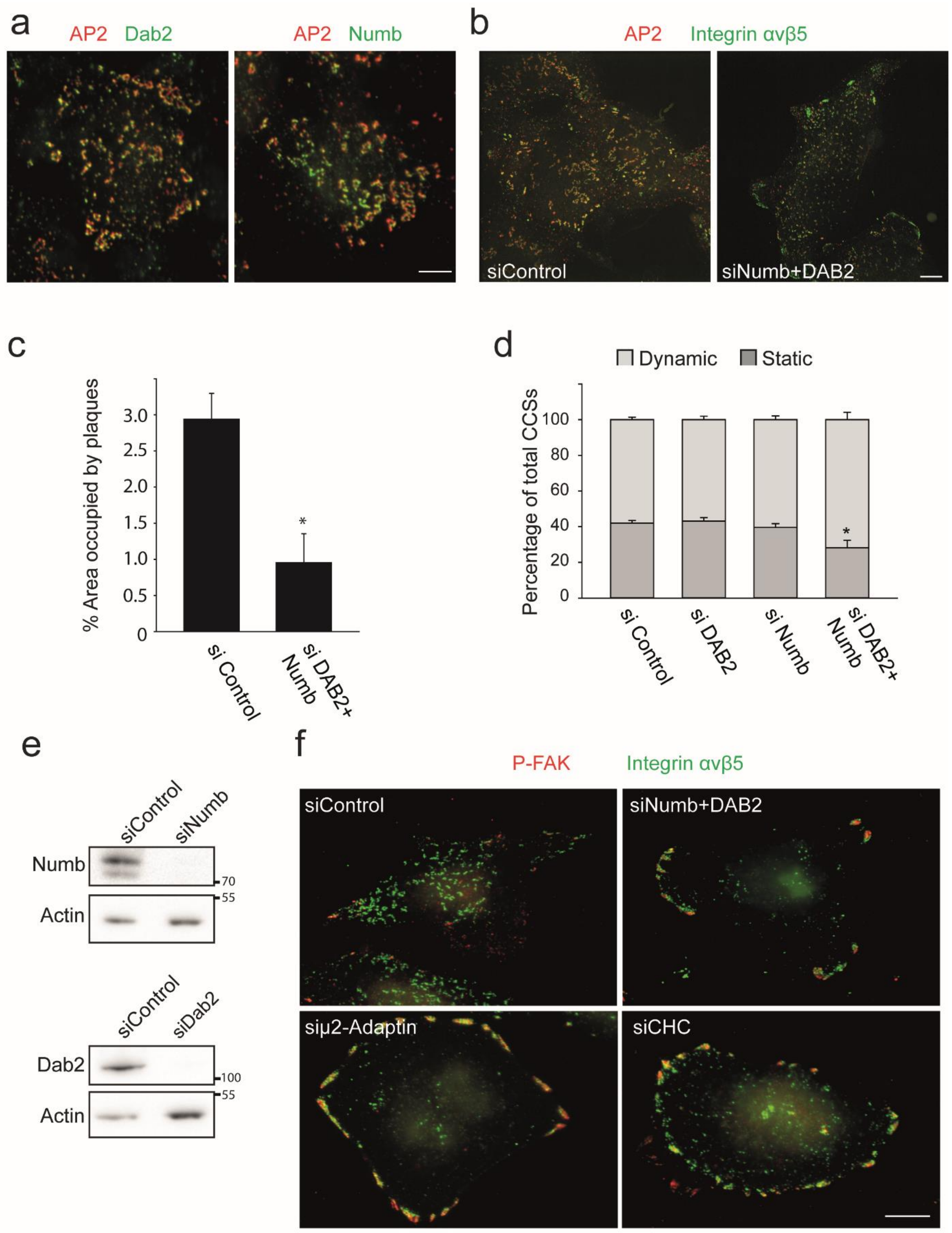

a
AP2 Dab2
AP2 Numb
b
AP2 Integrin αvβ5
siControl
siNumb+DAB2
c
% Area occupied by plaques
3.0
2.5
2.0
1.5
1.0
0.5
0
*
si Control
si DAB2+ Numb
d
Dynamic
Static
Percentage of total CCSs
100
80
60
40
20
0
*
si Control
si DAB2
si Numb
si DAB2+ Numb
e
siControl
siNumb
Numb
70
55
Actin
siControl
siDab2
Dab2
100
55
Actin
f
P-FAK Integrin αvβ5
siControl
siNumb+DAB2
siμ2-Adaptin
siCHC

SUPPLEMENTARY FIGURE 5. **Integrin αvβ5 shuttles between focal adhesions and clathrin-coated plaques**. **a**, HeLa cells were seeded on collagen-coated coverslips, fixed and immunostained for α-adaptin and Numb or Dab2. Scale bar: 5 µm. **b**, HeLa cells were seeded on collagen coated coverslips, fixed and immunostained for integrin αvβ5 and α-adaptin. Cells were then subjected to expansion microscopy. Scale bar: 5 µm. **c**, The percentage of ventral area occupied by plaques was measured in cells treated with control or Numb and Dab2 specific siRNAs. Expansion microscopy images were analysed. (* P<0.001, as assessed by One Way Analysis of Variance – ANOVA. N=3). **d**, HeLa cells genome edited to express GFP-tagged µ2-adaptin were transfected with the indicated siRNAs, then seeded on collagen-coated glass and imaged by spinning disk microscopy every 5s for 5 min. Results are expressed as mean ± SD (* *P*<0.001, as compared to siControl, as assessed by One Way Analysis of Variance – ANOVA. N=3). **e**, HeLa cells were treated with control, Numb or Dab2-specific siRNAs for 72h. Cells were then lysed and subjected to western blot analysis. **f**, HeLa cells treated with the indicated siRNAs for 48 h were seeded on collagen-coated glass and fixed 24h later in order to perform immunostaining for P-FAK (Tyr 397) and αvβ5 integrin.

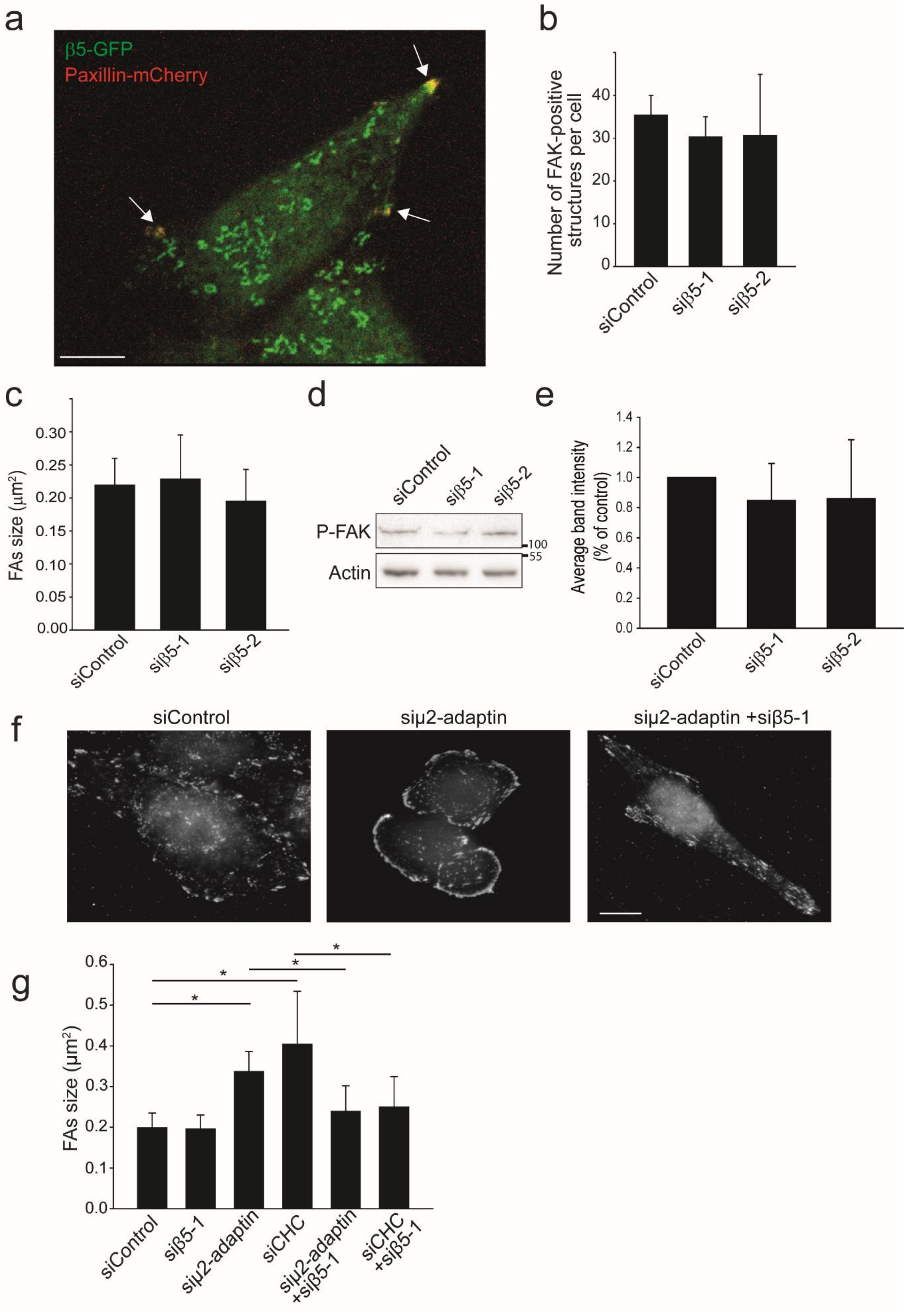

a
β5-GFP
Paxillin-mCherry
b
Number of FAK-positive structures per cell
0
10
20
30
40
siControl
siβ5-1
siβ5-2
c
FAs size (μm²)
0.00
0.05
0.10
0.15
0.20
0.25
0.30
siControl
siβ5-1
siβ5-2
d
siControl
siβ5-1
siβ5-2
P-FAK
Actin
100
55
e
Average band intensity (% of control)
0.0
0.2
0.4
0.6
0.8
1.0
1.2
1.4
siControl
siβ5-1
siβ5-2
f
siControl
siμ2-adaptin
siμ2-adaptin +siβ5-1
g
FAs size (μm²)
0.0
0.1
0.2
0.3
0.4
0.5
0.6
*
*
*
*
siControl
siβ5-1
siμ2-adaptin
siCHC
siμ2-adaptin +siβ5-1
siCHC +siβ5-1

SUPPLEMENTARY FIGURE 6. **Analysis of αvβ5 integrin localization and role at FAs. a**, HeLa cells expressing mCherry-tagged paxilllin and GFP-tagged β5 integrin were cultured onto collagen-coated glass and imaged 24h after seeding by spinning disk microscopy. Arrows point to paxillin- and β5-positive focal adhesions. Scale bar: 15 µm. **b**, Quantification of the average number of focal adhesion kinase (FAK)-positive FAs in HeLa cells treated with the indicated siRNA (One Way Analysis of Variance – ANOVA. N=3) **c**, Quantification of the average size of focal adhesion kinase (FAK)-positive FAs in HeLa cells treated with the indicated siRNA (One Way Analysis of Variance – ANOVA. N=3) **d**, HeLa cells treated with control or integrin-β5 specific siRNAs for 72 h were seeded on collagen coated glass and lysed in order to perform western blot analysis for P-FAK (representative image of three independent experiments) **e**, Densitometry analysis of bands obtained in Western-blots as in d (One Way Analysis of Variance – ANOVA. N=3) **f**, HeLa cells treated with the indicated siRNAs were seeded on collagen coated coverslips and subjected to immunostaining for Vinculin. Scale bar: 5 µm. **g**, Quantification of the average size of Vinculin-positive FAs in HeLa cells treated with the indicated siRNA (* P<0.001, as assessed by One Way Analysis of Variance – ANOVA. N=3). All data are expressed as mean ± SD.

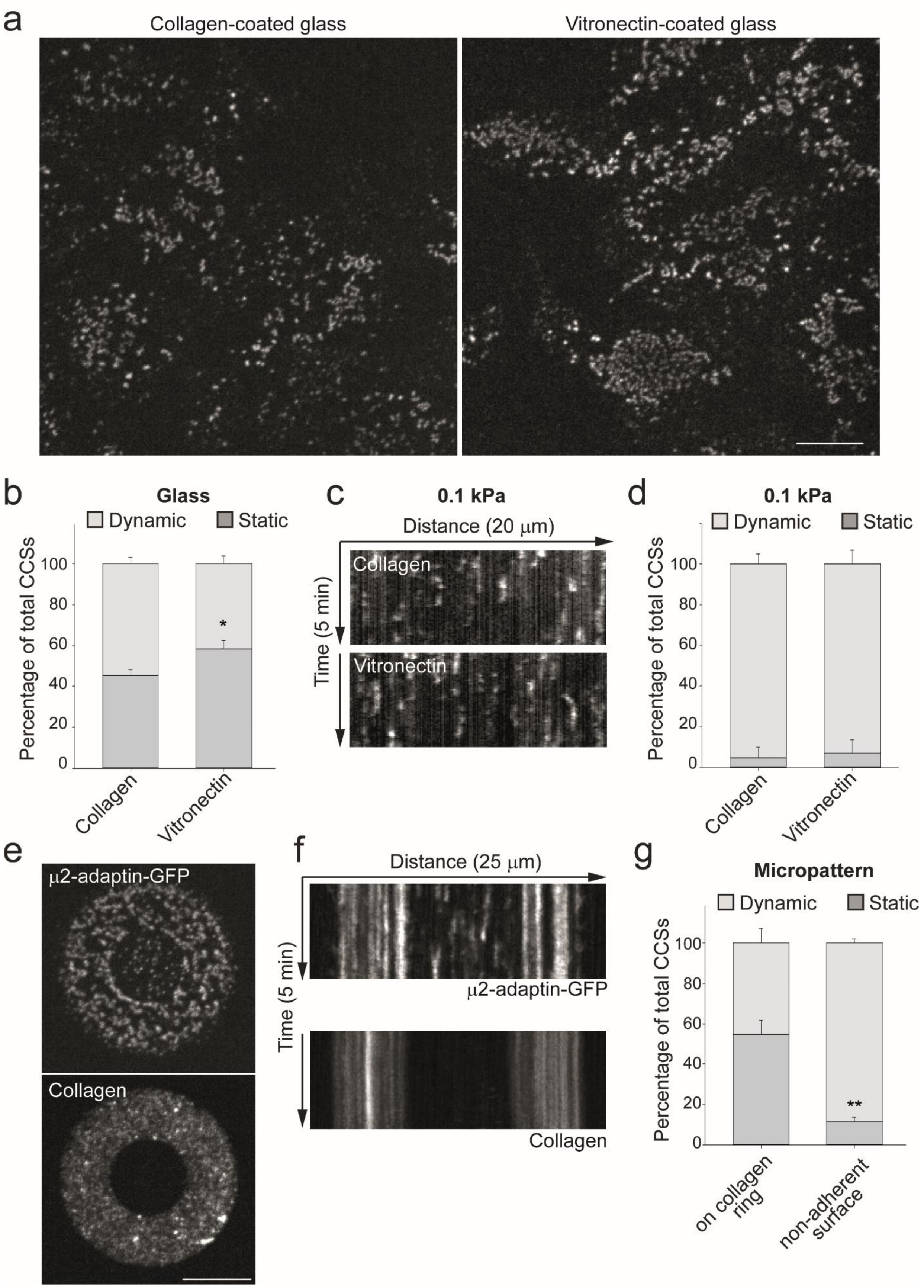
a
Collagen-coated glass
Vitronectin-coated glass
b
Glass
Dynamic
Static
Percentage of total CCSs
100
80
60
40
20
0
*
Collagen
Vitronectin
c
0.1 kPa
Distance (20 μm)
Time (5 min)
Collagen
Vitronectin
d
0.1 kPa
Dynamic
Static
Percentage of total CCSs
Collagen
Vitronectin
e
μ2-adaptin-GFP
Collagen
f
Distance (25 μm)
Time (5 min)
μ2-adaptin-GFP
Collagen
g
Micropattern
Dynamic
Static
Percentage of total CCSs
**
on collagen ring
non-adherent surface

SUPPLEMENTARY FIGURE 7. **Analysis of the role of the substrate in clathrin-coated plaque formation**. **a**, Genome-edited HeLa cells were seeded on collagen- or vitronectin-coated glass and imaged 24 h later by spinning disk microscopy. Scale bar: 15 µm. **b**, Quantification of the dynamics of CCSs in HeLa cells seeded on collagen- or vitronectin-coated glass (* $P<0.05$, as assessed by One Way Analysis of Variance – ANOVA. N=3). **c**, Kymographs showing CCS dynamics in genome-edited HeLa cells seeded onto collagen- or vitronectin-coated 0.1 kPa polyacrylamide gels, as indicated, and imaged 24 h later by spinning disk microscopy every 5s for 5 min. **d**, Quantification of the dynamics of CCSs in HeLa cells seeded on acrylamide gels coated with collagen or vitronectin as in c , (One Way Analysis of Variance – ANOVA. N=3). **e**, Genome-edited HeLa cells were seeded on Alexa546-collagen-coated ring-shaped micropatterns and imaged 24 h later by spinning disk microscopy. Scale bar: 15 µm. **f**, Kymographs showing CCS dynamics in a HeLa cell as in e and imaged by spinning disk microscopy every 5s for 5 min. **g**, Quantification of the dynamics of CCSs observed as in e and f and located on the collagen-coated adherent pattern or on the non-adherent region, as indicated (** $P<0.001$, as assessed by One Way Analysis of Variance – ANOVA. N=3). All data are expressed as mean ± SD.

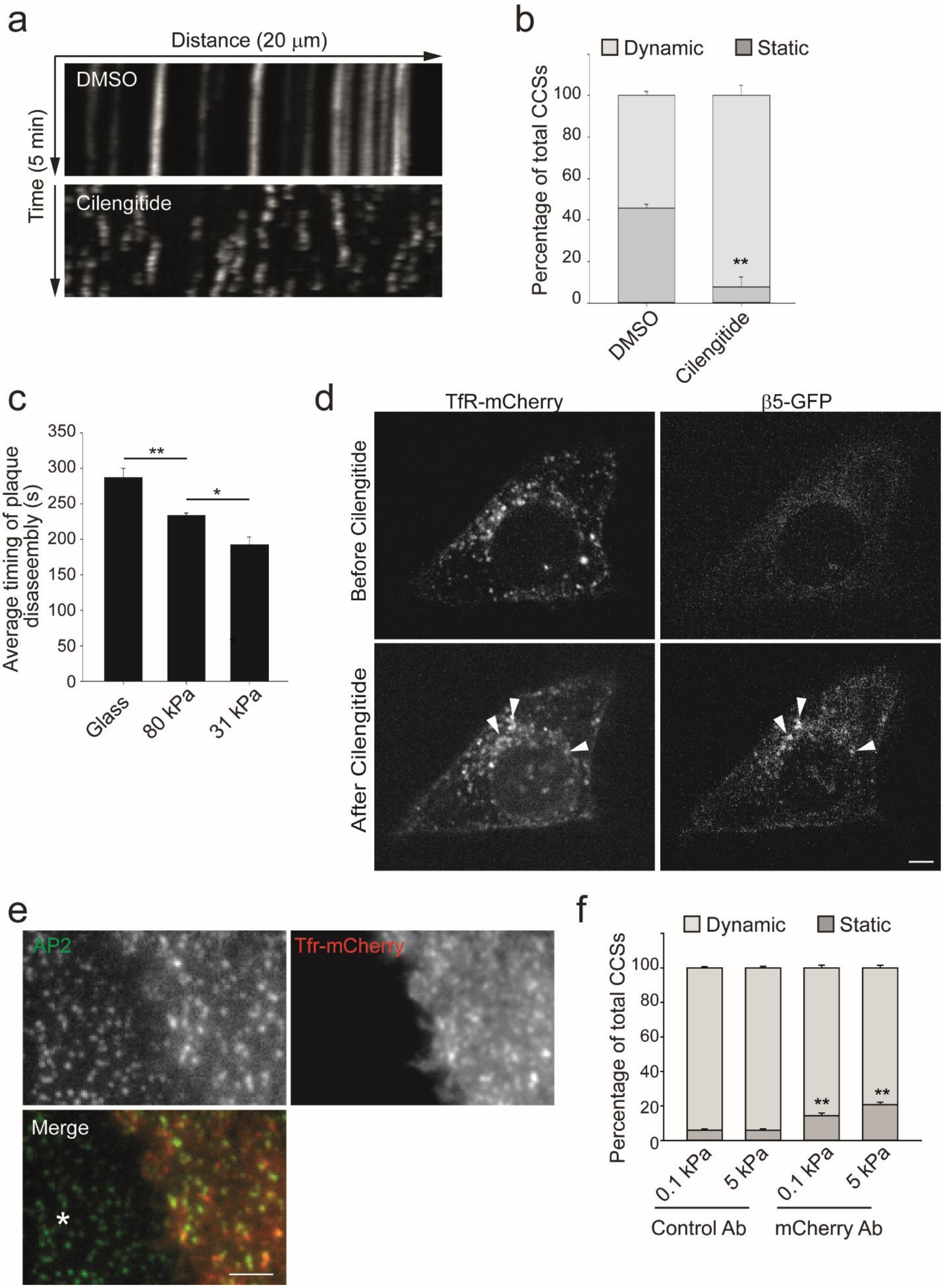
a
Distance (20 μm)
Time (5 min)
DMSO
Cilengitide
b
Dynamic
Static
Percentage of total CCSs
100
80
60
40
20
0
**
DMSO
Cilengitide
c
Average timing of plaque disaseembly (s)
350
300
250
200
150
100
50
0
**
*
Glass
80 kPa
31 kPa
d
TfR-mCherry
β5-GFP
Before Cilengitide
After Cilengitide
e
AP2
Tfr-mCherry
Merge
*
f
Dynamic
Static
Percentage of total CCSs
100
80
60
40
20
0
**
**
0.1 kPa
5 kPa
0.1 kPa
5 kPa
Control Ab
mCherry Ab

SUPPLEMENTARY FIGURE 8. **Analysis of the effect of Cilengitide on plaques disassembly**. **a**, Kymographs showing CCS dynamics in genome-edited HeLa cells expressing endogenous mCherry-tagged μ2-adaptin. Cells were seeded on collagen-coated glass and treated 24 h later with DMSO or 10 μM Cilengitide for 30 min before being imaged by spinning disk microscopy every 5 sec for 5 min. **b**, quantification of the dynamics of CCSs observed as in a (* $P<0.001$, as assessed by One Way Analysis of Variance – ANOVA. N=3). **c**, Genome-edited HeLa cells expressing endogenous mCherry-tagged μ2-adaptin were seeded on collagen-coated glass or on collagen-coated polyacrylamide gels of the indicated stiffness and imaged 24 h later by spinning disk microscopy every 10 sec for 10 min. 10 μM Cilengitide was added during acquisition and the average time needed to lose plaques was measured. Measurements were performed on 100 plaques per condition from 9 cells from three independent experiments (* $P<0.005$, as assessed by One Way Analysis of Variance – ANOVA). **d**, HeLa cells were transfected with plasmids encoding for TfR-mCherry and Integrin β5-GFP and the intracellular content was analyzed by spinning disk microscopy before and 30 min after cilengitide addition. Arrows point to vesicles positive for Integrin β5-GFP-and TfR-mCherry. Scale Bar: 5 μm. **e**, Genome edited HeLa cells expressing endogenous GFP tagged μ2-adaptin were treated with integrin β5 specific siRNAs. Cells were then transfected with a plasmid encoding TfR-mCherry and seeded on glass coated with anti-mCherry antibodies. Cells were then imaged with TIRF microscopy. Scale bar: 2 μm. * untransfected cell. **f**, CCSs dynamics in genome edited HeLa cells transfected with TfR-mCherry and seeded on acrylamide gels of the indicated stiffness coated with control or anti-mCherry antibodies and imaged by spinning disk microscopy every 5s for 5 min (** $P<0.001$, as assessed by One Way Analysis of Variance – ANOVA. N=3). All data are expressed as mean ± SD.

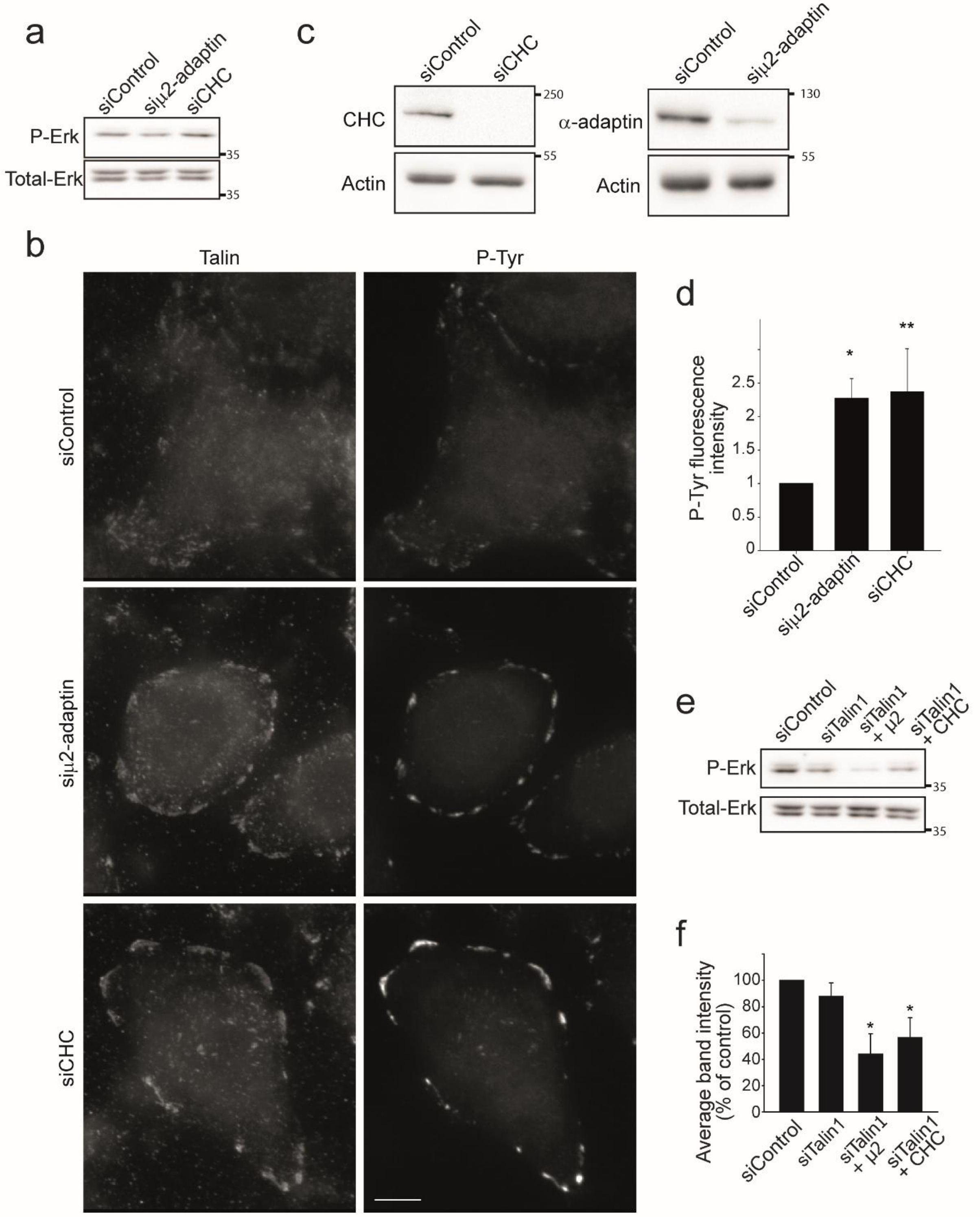

a
siControl
siμ2-adaptin
siCHC
P-Erk
Total-Erk
35
35
c
siControl
siCHC
CHC
Actin
250
55
siControl
siμ2-adaptin
α-adaptin
Actin
130
55
b
Talin
P-Tyr
siControl
siμ2-adaptin
siCHC
d
P-Tyr fluorescence intensity
0
0.5
1
1.5
2
2.5
*
**
siControl
siμ2-adaptin
siCHC
e
siControl
siTalin1
siTalin1 + μ2
siTalin1 + CHC
P-Erk
Total-Erk
35
35
f
Average band intensity (% of control)
0
20
40
60
80
100
*
*
siControl
siTalin1
siTalin1 + μ2
siTalin1 + CHC

SUPPLEMENTARY FIGURE 9. **AP-2 or CHC depletion modulate FAs size and their phospho-tyrosine contents**. **a**, HeLa cells treated with the indicated siRNAs were seeded on collagen coated glass and Erk activation was analysed by western blot (representative image of three independent experiments) **b**, HeLa cells transfected with the indicated siRNAs were seeded onto collagen-coated glass and fixed 24 hours later before being stained for Talin and phospho-tyrosines (P-Tyr), as indicated. Scale bar: 10 μm. **c**, Western blot analysis of CHC expression (left) and α-adaptin (right) in HeLa cells treated with the indicated siRNAs. Actin was used as a loading control. **d**, Quantification of P-Tyr enrichment at the periphery of HeLa cells treated with the indicated siRNA and stained as in b (* $P<0.01$ as compared to siControl, as assessed by One Way Analysis of Variance – ANOVA. N=3). **e**, HeLa cells treated with the indicated siRNAs were seeded on collagen-coated glass and Erk activation was analyzed by western blot (representative image of four independent experiments) **f**, Densitometry analysis of bands obtained in Western-blots as in E (* $P<0.05$ as compared to siTalin1, as assessed by One Way Analysis of Variance – ANOVA. N=4). All data are expressed as mean ± SD.

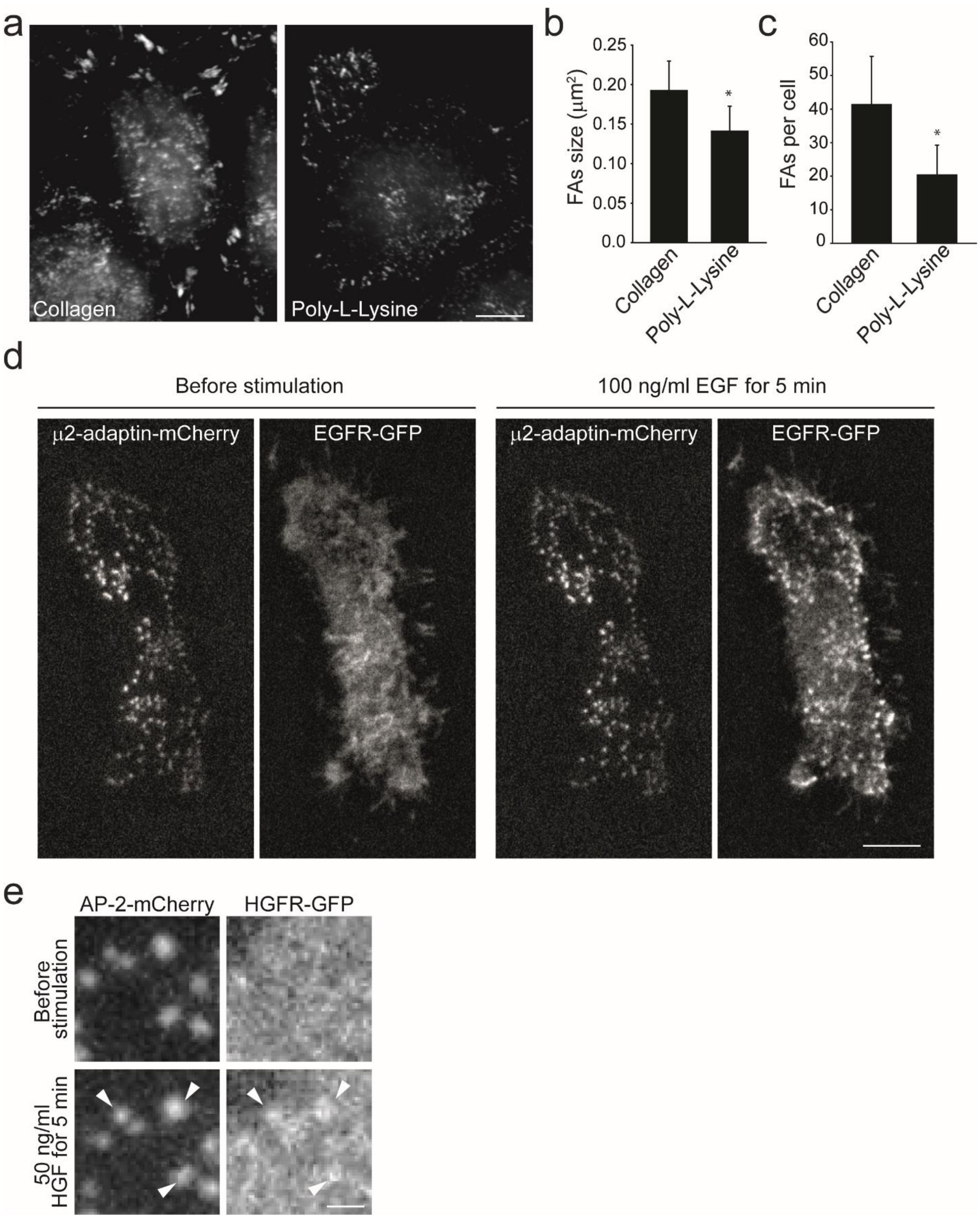

a
Collagen
Poly-L-Lysine
b
FAs size (μm2)
0.25
0.20
0.15
0.10
0.05
0.0
*
Collagen
Poly-L-Lysine
c
FAs per cell
60
50
40
30
20
10
0
*
Collagen
Poly-L-Lysine
d
Before stimulation
100 ng/ml EGF for 5 min
μ2-adaptin-mCherry
EGFR-GFP
μ2-adaptin-mCherry
EGFR-GFP
e
AP-2-mCherry
HGFR-GFP
Before stimulation
50 ng/ml HGF for 5 min

SUPPLEMENTARY FIGURE 10. **Analysis of receptor-clustering at plaques**. **a**, HeLa cells were seeded on collagen-coated or anti-mCherry antibodies/Poly-L-Lysine treated glass, then fixed and subjected to immunostaining for vinculin. Scale bar: 5 µm. **b**, Quantification of the average size of Vinculin-positive FAs as in the images in a (* P<0.001, as assessed by One Way Analysis of Variance – ANOVA. N=3). **c**, Quantification of the average number of Vinculin-positive FAs as in the images in a (* P<0.001, as assessed by One Way Analysis of Variance – ANOVA. N=3). **d**, HeLa cells transfected with plasmids encoding µ2-adaptin-mCherry and EGFR-GFP were plated on collagen-coated glass-bottom dishes. The following day, cells were serum-starved for 2 hours prior to image them by TIRF microscopy. During image acquisition, cells were stimulated with 10 ng/ml EGF for 5 min. Scale bar: 10 µm. **e**, Genome edited HeLa cells expressing µ2-adaptin-mCherry were transfected with a plasmid encoding HGFR-GFP, seeded on collagen-coated glass, and serum-starved the next day for 2 hours prior to image them by TIRF microscopy. During image acquisition, cells were stimulated with 50 ng/ml HGF for 10 minutes. Arrows point to HGFR-GFP colocalizing with µ2-adaptin-mCherry. Scale bar: 0.5 µm. All data are expressed as mean ± SD.

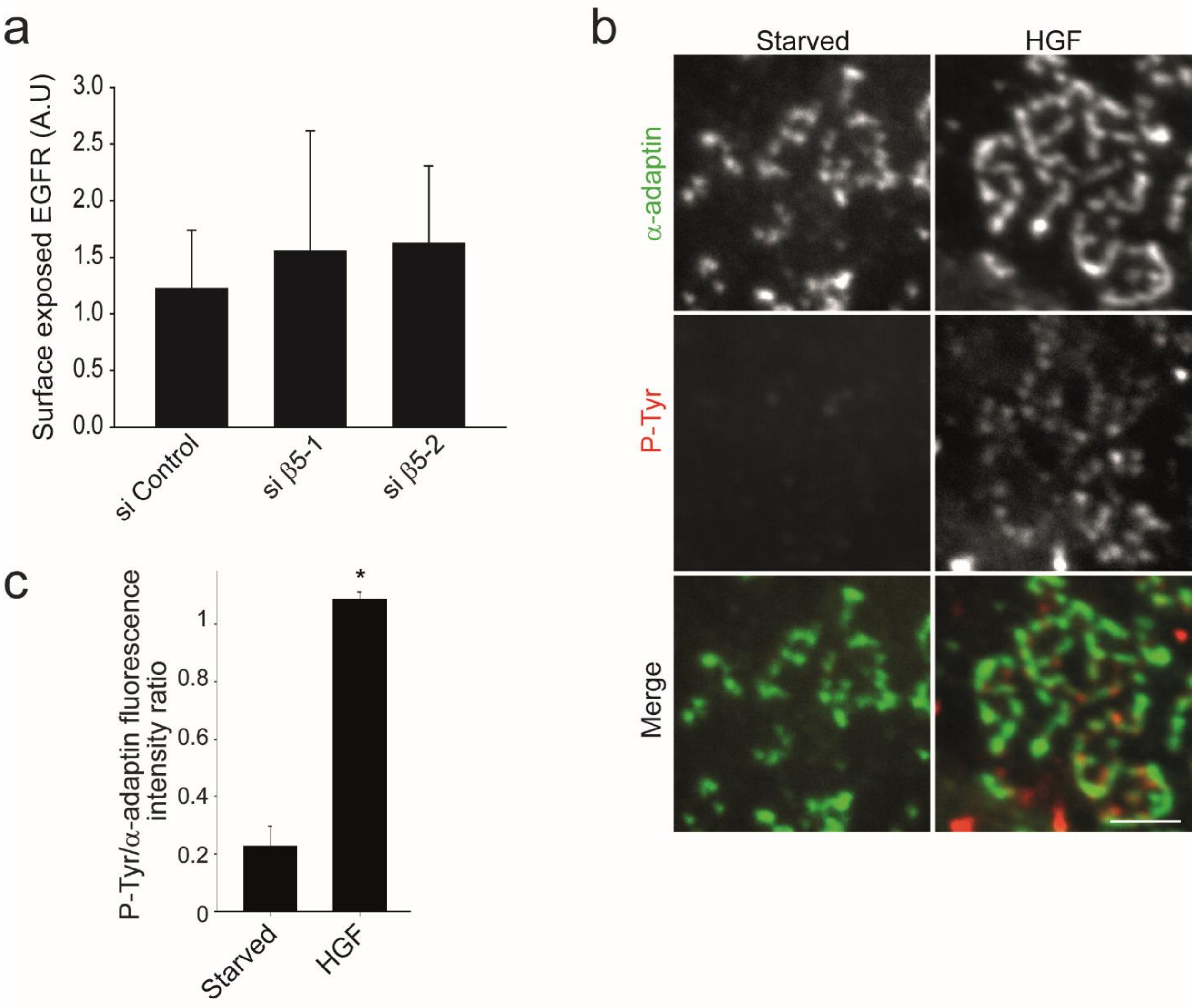

SUPPLEMENTARY FIGURE 11. **Analysis of receptor-dependent signalling at plaques**. **a**, HeLa cells treated with the indicated siRNAs were seeded on collagen coated coverslips. The following day, cells were incubated on ice with 200 ng/ml Alexa488-labelled EGF at 4°C for 1 hour. Cells were then fixed and images were taken in order to evaluate the amount of cell surface EGFR (One Way Analysis of Variance – ANOVA. N=3) **b**, HeLa cells were seeded on collagen coated glass, and serum-starved the next day for 4 hours before stimulating them with 50 ng/ml HGF for 5 minutes. Cells were then fixed and stained for α-adaptin and phospho-tyrosines (P-Tyr). Scale bar: 2 µm. **c**, Quantification of phospho-tyrosines enrichment in HGF-stimulated cells versus serum-starved cells (* *P*<0.01, as assessed by Student's T-test. N=3). All data are expressed as mean ± SD.

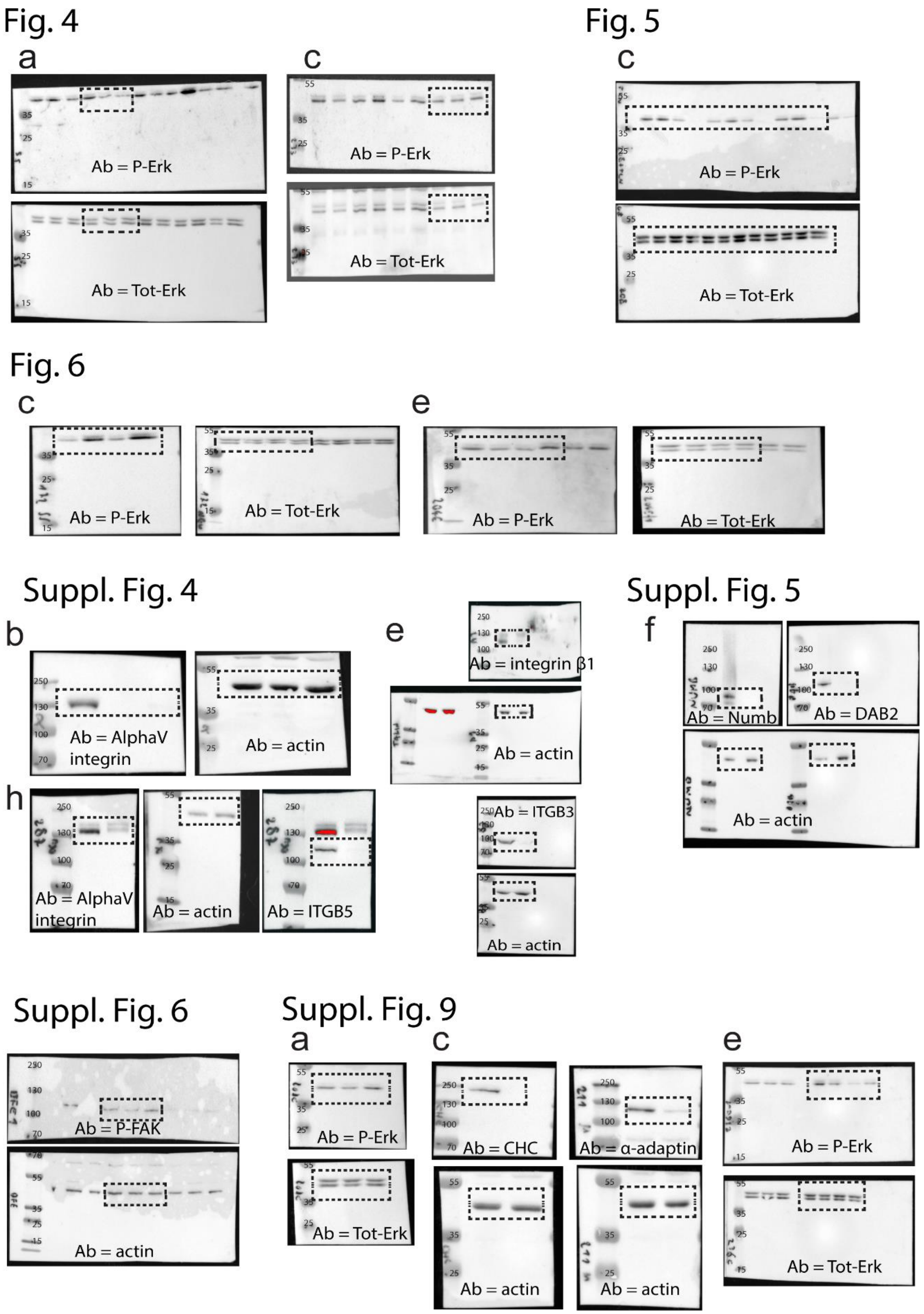

SUPPLEMENTARY FIGURE 12. **Uncropped western blot images**.
