# Supplementary material for: Frustrated endocytosis controls contractility-independent mechanotransduction at clathrin-coated structures
Source: Nat Commun. 2018 Sep 20;9:3825. doi: 10.1038/s41467-018-06367-y (PMC6148028; doi:10.1038/s41467-018-06367-y)
Supplement: Supplementary file 3 — Description of Additional Supplementary Files [file 41467_2018_6367_MOESM3_ESM.pdf]

Description of Additional Supplementary Files

Supplementary Movie 1

Description: CCS dynamics is regulated by substrate rigidity. HeLa cells genome-edited to express endogenous GFP-tagged μ2-adaptin were seeded onto collagen-coated polyacrylamide gels of the indicated stiffness. The next day, cells were imaged by spinning disk microscopy every 5s for 5 min. Scale bar: 5 µm. Time is indicated in seconds.

Supplementary Movie 2

Description: β5-integrin is required for plaque formation. HeLa cells genome-edited to express endogenous GFP-tagged μ2-adaptin were treated with the control (left panel) or β5-specific (right panel) siRNAs, as indicated. 48 h later, cells were seeded onto collagen-coated glass-bottom dishes and imaged the next day by spinning disk microscopy every 5s for 5 min. Scale bar: 5 µm. Time is indicated in seconds.

Supplementary Movie 3

Description: Trypsin-induced plaque disassembly is accompanied by CCPs budding marked by auxillin bursts. HeLa cells genome-edited to express endogenous mCherry-tagged μ2-adaptin (red) were transfected with a plasmid encoding for GFP-tagged Auxillin (green) and seeded onto collagen-coated glass-bottom dish. The next day, cells were briefly washed in PBS before being incubated with trypsin (see materials and methods) and imaged by spinning disk microscopy every 2s for 3 min. Scale bar: 2 µm. Time is indicated in seconds.

Supplementary Movie 4

Description: Cilengitide treatment prevents plaque formation. HeLa cells genome-edited to express endogenous GFP-tagged μ2-adaptin were seeded onto collagen-coated glass-bottom dishes. The next day, cells were treated with 10 μM Cilengitide for 30 min and imaged by spinning disk microscopy every 5s for 5 min. Scale bar: 5 μm. Time is indicated in seconds.

Supplementary Movie 5

Description: Dynamics of Cilengitide-induced plaque disassembly. HeLa cells genome-edited to express endogenous mCherry-tagged μ2-adaptin were seeded onto collagen-coated glass-bottom dishes. The next day, cells were treated with 10 μM Cilengitide and imaged immediately by spinning disk microscopy every 10s for 10 min. Scale bar: 5 μm. Time is indicated in seconds.

Supplementary Movie 6

Description: Immobilizing the TfR receptor stalls CCSs dynamics. HeLa cells genome-edited to express endogenous GFP-tagged μ2-adaptin (green) were transfected with a plasmid encoding for mCherry-TfR (red) and seeded onto anti-mCherry antibody-coated glass-bottom (see materials and methods). The next day, cells were imaged by spinning disk microscopy every 5s for 5 min. Scale bar: 5 μm. Time is indicated in seconds.
